# Supplementary material for: Cell Marker Accordion: interpretable single-cell and spatial omics annotation in health and disease
Source: Nat Commun. 2025 Jul 7;16:5399. doi: 10.1038/s41467-025-60900-4 (PMC12234662; doi:10.1038/s41467-025-60900-4)
Supplement: Supplementary file 1 — Supplementary Information [file 41467_2025_60900_MOESM1_ESM.pdf]

SUPPLEMENTARY MATERIALS

Cell Marker Accordion: interpretable single-cell and spatial omics annotation in health and disease

Emma Busarello[1,*,✉], Giulia Biancon[2,3,*], Ilaria Cimignolo[1], Fabio Lauria[4], Zuhairia Ibnat[1], Christian Ramirez[1], Gabriele Tomè[1,4], Marianna Ciuffreda[1], Giorgia Bucciarelli[1], Alessandro Pilli[1], Stefano Maria Marino[1], Vittorio Bontempi[5], Federica Ress[6], Kristin R. Aass[7], Jennifer VanOudenhove[2], Luca Tiberi[6], Maria Caterina Mione[5], Therese Standal[7], Paolo Macchi[8], Gabriella Viero[4], Stephanie Halene[2,9,✉] and Toma Tebaldi[1,2,✉]

1 Laboratory of RNA and Disease Data Science, Department of Cellular, Computational and Integrative Biology (CIBIO), University of Trento, Trento, Italy

2 Section of Hematology, Department of Internal Medicine, Yale Comprehensive Cancer Center, Yale University School of Medicine, New Haven, CT, USA

3 Hematology Unit, Fondazione IRCCS Ca' Granda Ospedale Maggiore Policlinico, Milan, Italy

4 Institute of Biophysics, CNR Unit at Trento, Italy

5 Laboratory of Experimental Cancer Biology, Department of Cellular, Computational and Integrative Biology (CIBIO), University of Trento, Trento, Italy

6 *Armenise-Harvard Laboratory of Brain Disorders and Cancer, Department of Cellular, Computational and Integrative Biology (CIBIO), University of Trento, Trento, Italy*

7 Department of Clinical and Molecular Medicine, Norwegian University of Science and Technology (NTNU), Trondheim, Norway

8 Laboratory of Molecular and Cellular Neurobiology, Department of Cellular, Computational and Integrative Biology (CIBIO), University of Trento, Trento, Italy

9 Department of Pathology, Yale University School of Medicine, New Haven, CT, USA

SUPPLEMENTARY FIGURES

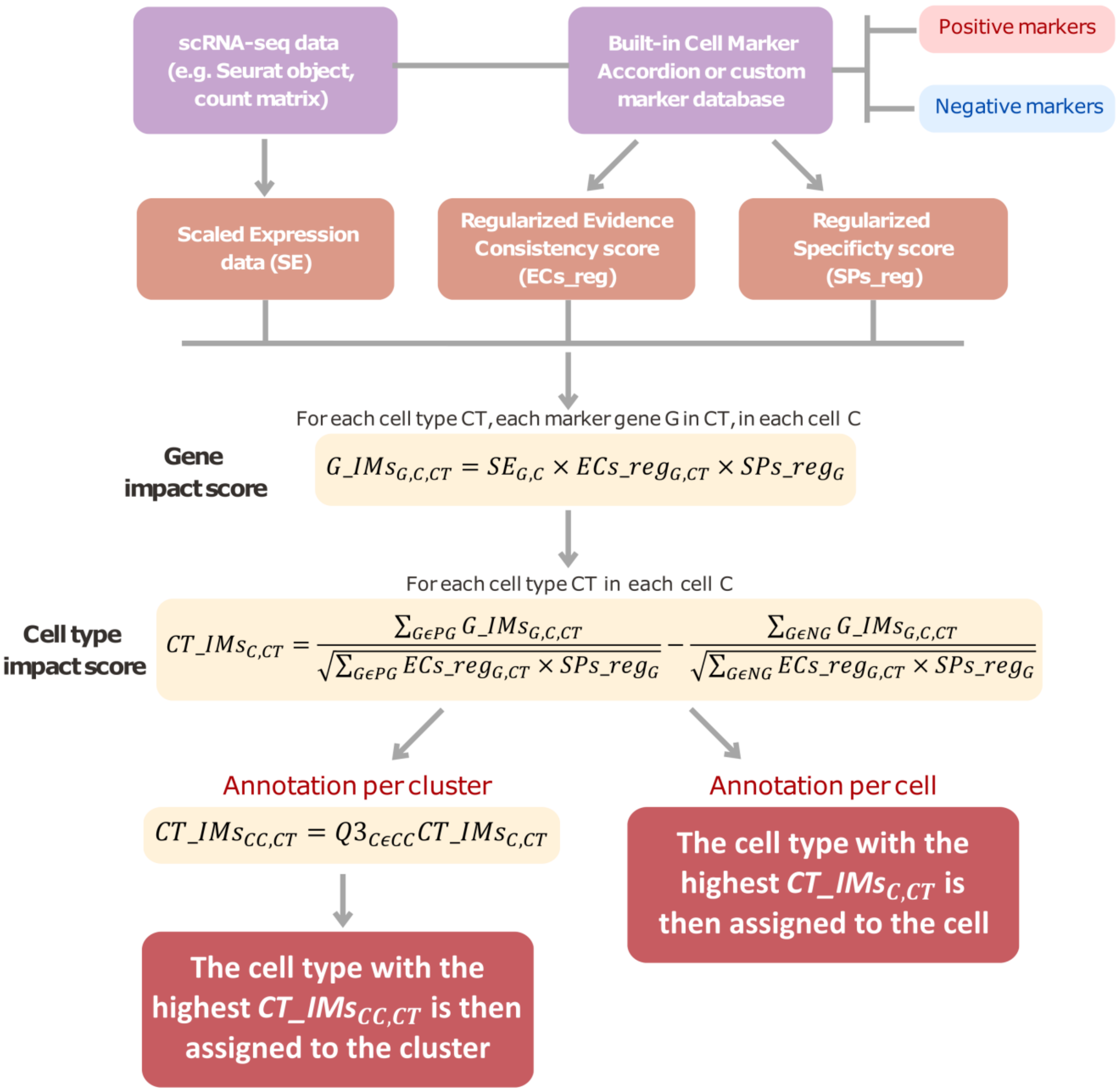

Supplementary Fig.1: The Cell Marker Accordion: annotation and scoring workflow. *The figure outlines the Cell Marker Accordion annotation workflow: the necessary input, the main algorithmic steps and the primary annotation output (see* ***Methods****). SE: scaled expression data (e.g. Z-score); ECs: evidence consistency score; ECs_reg: regularized evidence consistency score; SPs: specificity score; SPs_reg: regularized specificity score; CT: cell type; G: marker gene; G_IMs: gene impact score, calculated for each cell type CT, each marker gene G, in each cell; PG: positive marker gene for cell type CT; NG: negative marker gene for cell type CT; CT_IMs: cell type impact score, calculated for each cell type CT in each cell; CC: cell cluster;* CT_IMs_{CC}*: cell cluster cell type impact score.*

Related to Fig.2

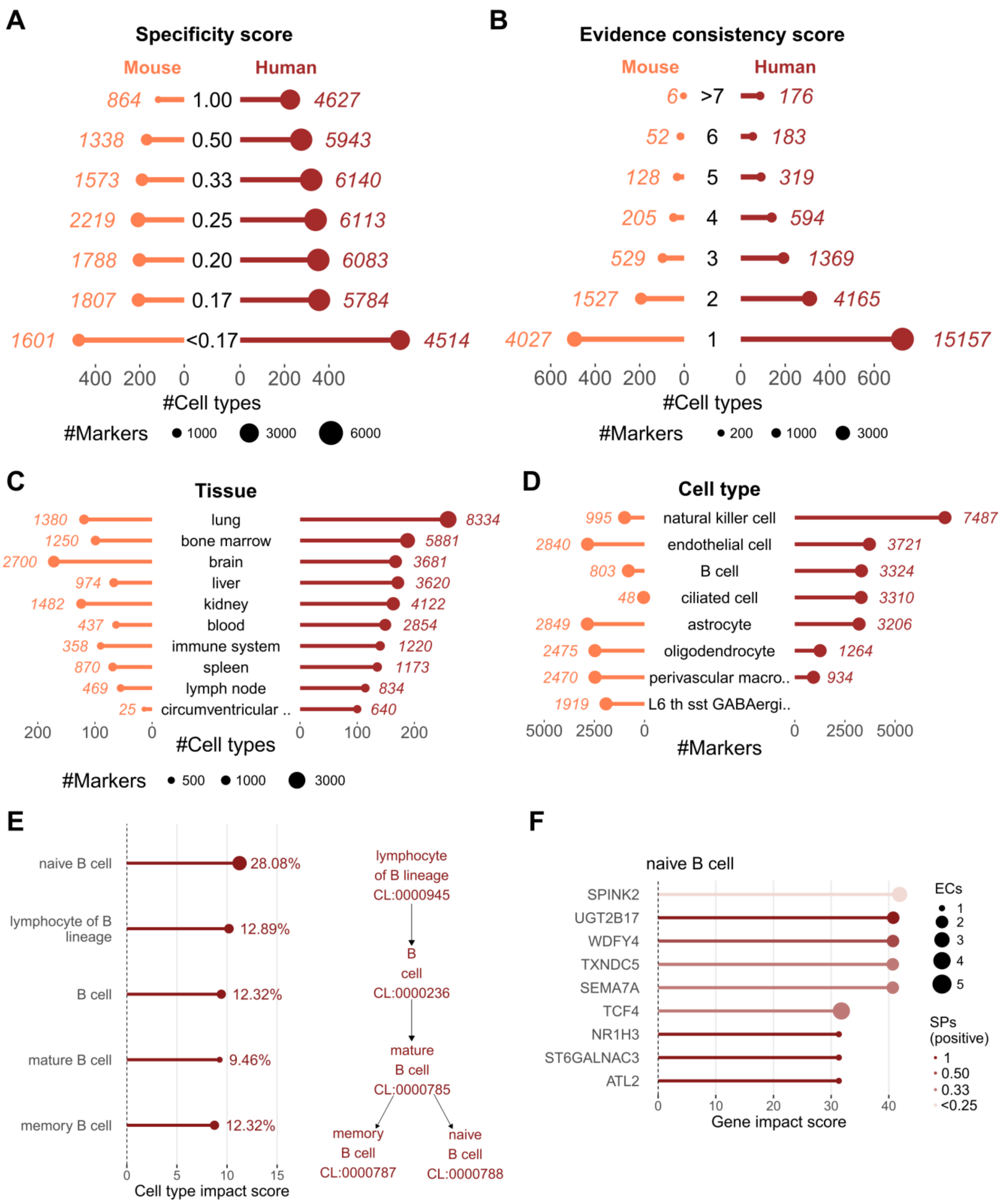

***Supplementary Fig. 2: The Cell Marker Accordion: scores and detailed annotation results. A** Number of cell types, reported on x axis, and number of markers, corresponding to dot size, for each specificity value in mouse (orange bars) and human (red bars). **B** Number of cell types, reported on x axis, and number of markers, corresponding to dot size, for each evidence consistency score value in mouse (orange bars) and human (red bars). **C** Cell Marker Accordion tissues with the highest number of markers for mouse (orange bars) and human (red bars). **D** Cell Marker Accordion cell types with the highest number of markers for mouse (orange bars) and human (red bars). **E** Example of cell types ranking output. On the left side, the top 5 cell types that compete for the annotation of*

the same cluster are ordered according to their impact score. The percentage of cells labelled as the corresponding cell types is also shown. On the right side, the ontology tree of the top 5 cell types is represented. ***F*** *Example of markers ranking output for a particular cluster. The most influential markers which drive the identification of the associated cell type, in this case, "naive B cell", are ordered according to their impact score. Dot size represents the evidence consistency score, and colors refer to marker specificity.*

Related to Fig.2

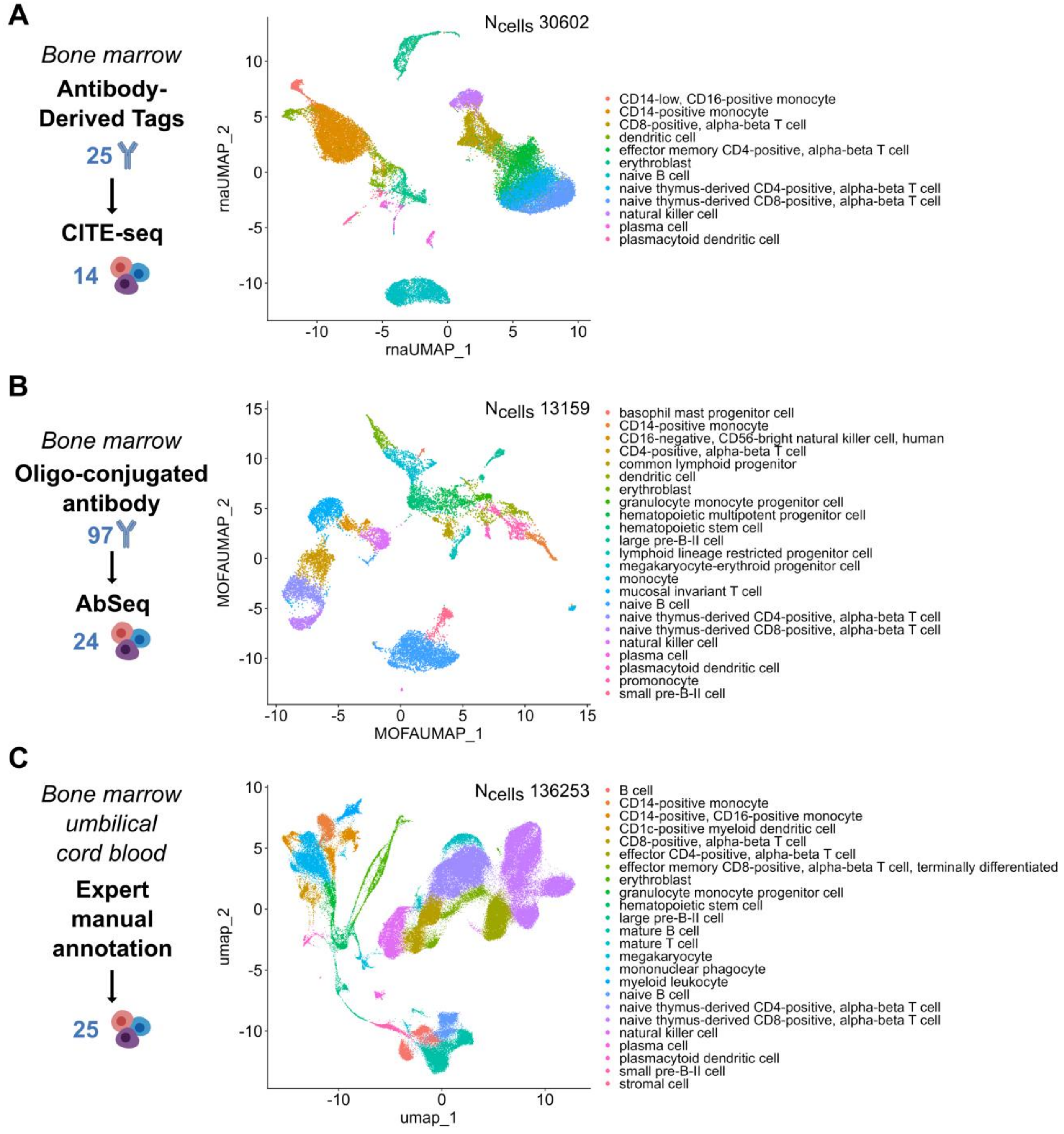

Supplementary Fig.3: The Cell Marker Accordion annotation of cell types in complex single-cell multiomics. *Annotation with the Cell Marker Accordion of multi-omics single-cell datasets.* ***A*** *Human bone marrow dataset was obtained with a CITE-seq multi-modal approach (25 barcoded antibodies were used to quantify surface proteins and identify 14 different cell types, considered as the ground truth). Populations annotated by the Accordion are color-coded in the UMAP.* ***B*** *Human bone marrow dataset was obtained with an Ab-seq multi-modal approach (97 barcoded antibodies were used to quantify surface proteins and identify 24 different cell types, which is considered the ground truth). Populations annotated by the Accordion are color-coded in the UMAP.* ***C*** *Single-cell RNA-seq dataset of human cells from bone marrow and umbilical cord blood. Expert-based manual annotation identified 25 different cell types, considered as the ground truth.*

Related to Fig.3

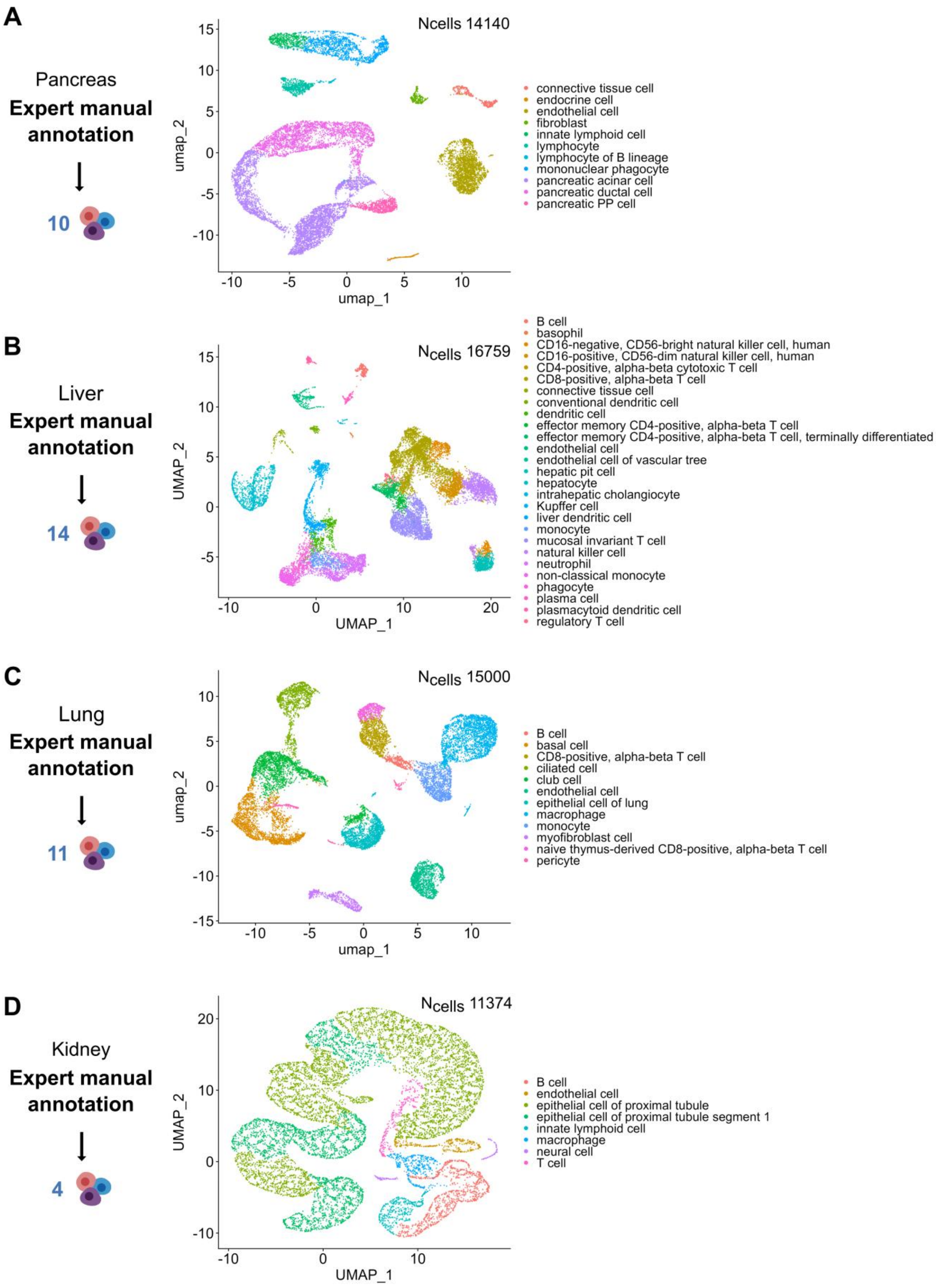

Supplementary Fig.4: Cell Marker Accordion annotation of cell types in multiple tissues. *Annotation with the Cell Marker Accordion of single-cell datasets in multiple tissues **A** Single-cell*

RNA-seq dataset of human cells from pancreas. Expert based manual annotation identified 10 different cell types, considered as the ground truth. ***B*** *Single-cell RNA-seq dataset of human cells from liver. Expert based manual annotation identified 14 different cell types, considered as the ground truth. Populations annotated by the Accordion are color-coded in the UMAP.* ***C*** *Single-cell RNA-seq dataset of human cells from lung. Expert based manual annotation identified 11 different cell types, considered as the ground truth. Populations annotated by the Accordion are color-coded in the UMAP.* ***D*** *Single-cell RNA-seq dataset of human cells from kidney. Expert based manual annotation identified 4 different cell types, considered as the ground truth. Populations annotated by the Accordion are color-coded in the UMAP.*

Related to Fig.3

A

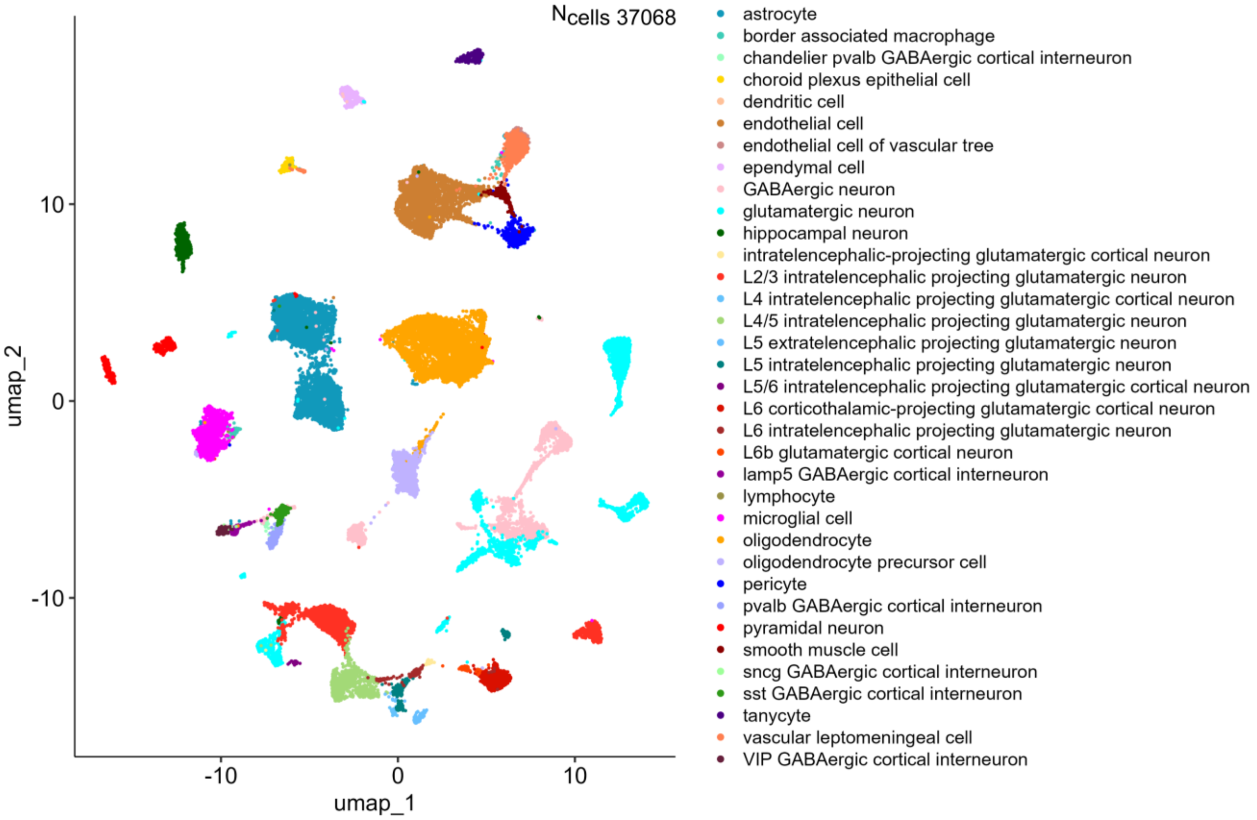

Supplementary Fig.5: Mouse brain populations in spatial transcriptomic dataset. A *UMAP plot based on the transcriptional profile of each cell, with colors based on the original published annotation, mapped to Cell Ontology terms.*
Related to Fig.4

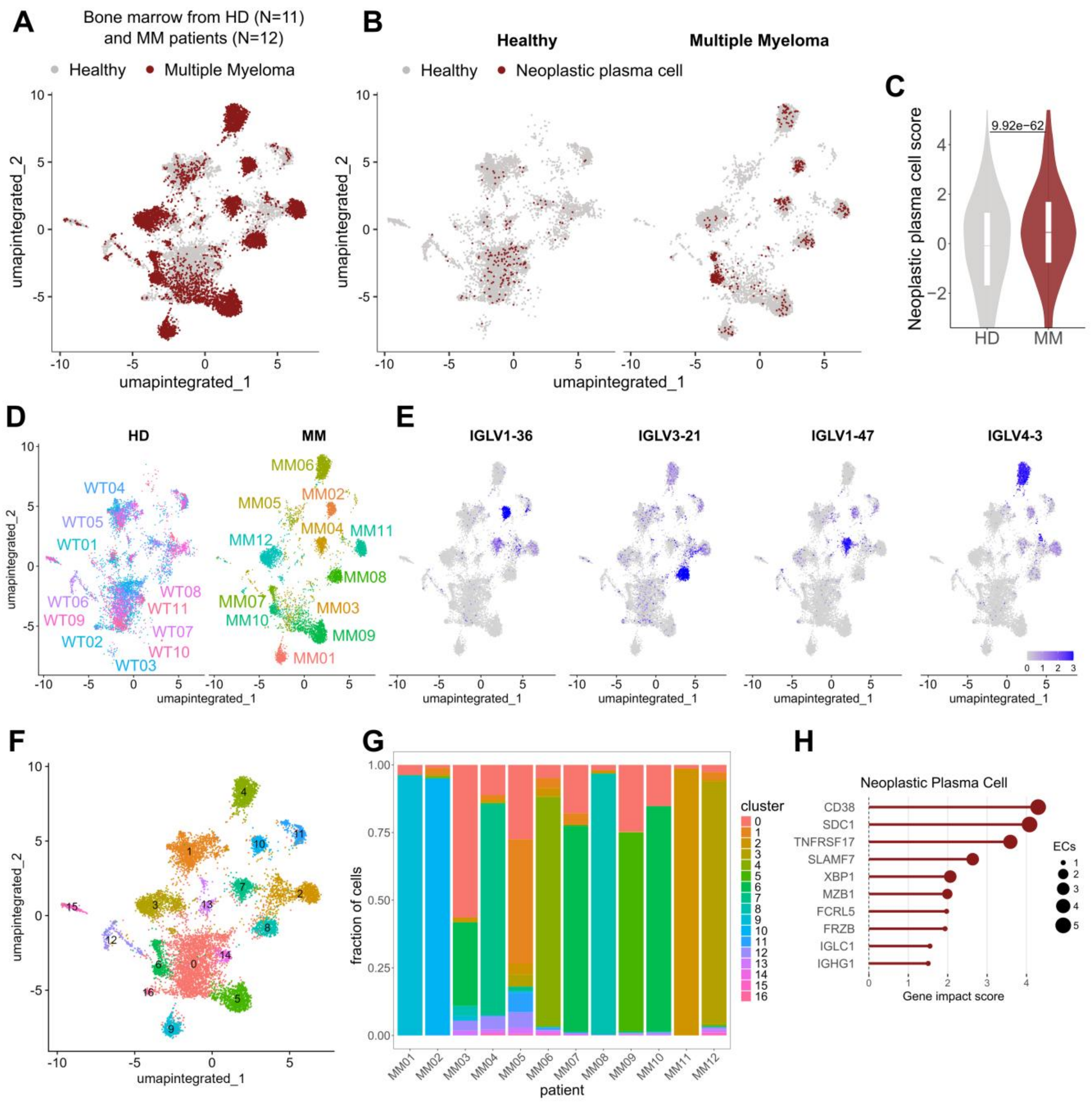

Supplementary Fig. 6: The Cell Marker Accordion identifies myeloma plasma cells subtypes in multiple myeloma patients. ***A*** *Human bone marrow cells from healthy donors (HD) and multiple myeloma (MM) patients, for which bone marrow plasma cells were single-cell sorted by FACS and then sequenced.* ***B*** *Identification of neoplastic plasma cells with cell resolution. Cells are colored according to the neoplastic plasma cell score.* ***C*** *Distribution of neoplastic plasma cell scores. A significant increase is observed in MM patients. The box plots represent the median as the central line, while the lower and upper hinges correspond to the first and third quartiles (25th and 75th percentiles). Whiskers extend to the smallest and largest values within 1.5 times the interquartile range from the lower and upper quartiles, respectively. One-tailed Wilcoxon Rank Sum test was used, P-value is displayed.* ***D*** *UMAP showing HDs and MM patients cells. Cells from the same patients are color-coded.* ***E*** *Expression of Immunoglobulin variable region (IGVL) genes. Cells are colored according to gene expression levels.* ***F*** *UMAP visualization of cells of HD and MM patients, with clusters color-coded and labelled.* ***G*** *Cluster composition in each MM patient.* ***H*** *Marker genes*

with the highest impact in defining neoplastic plasma cells from MM patients. One-tailed Wilcoxon Rank Sum test was used for panel ***C****. P-value is displayed.*

Related to Fig.5

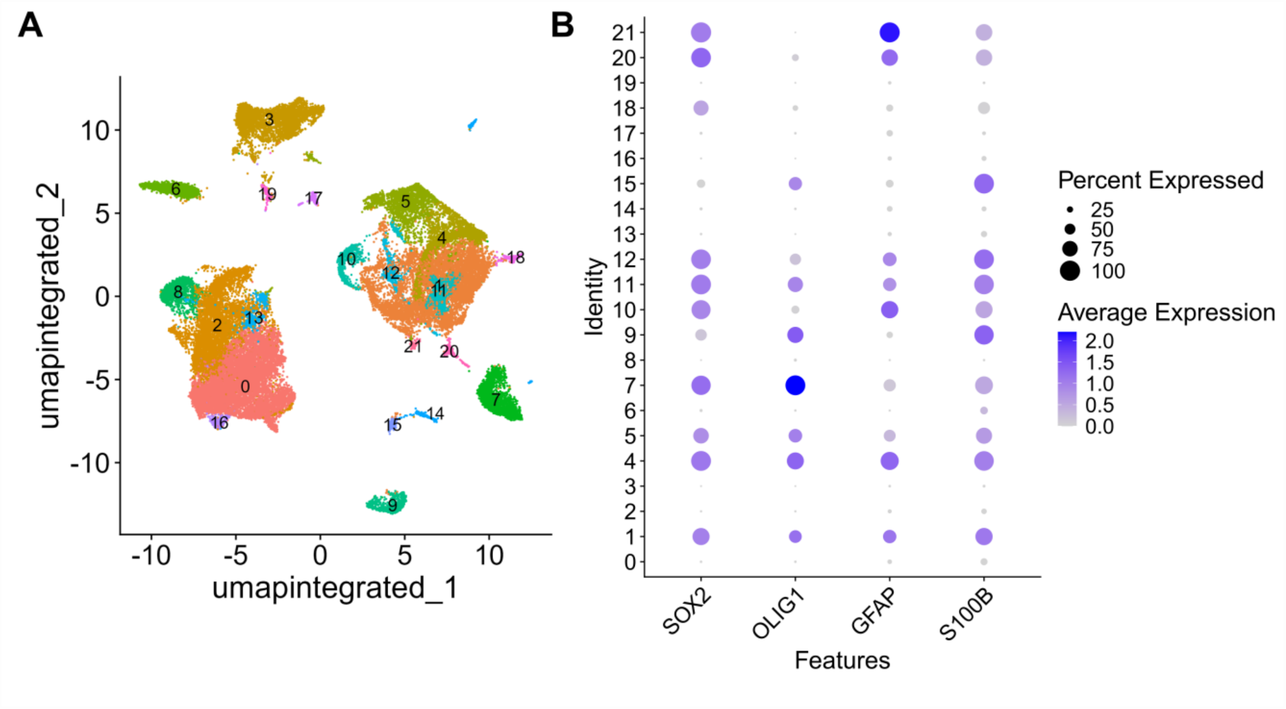

***Supplementary Fig.7: Clusters of tumoral cells in glioblastoma patients. A** UMAP visualization of cell clustering from glioblastoma patient samples, with clusters color-coded and labelled. **B** Expression levels of tumor markers across the identified clusters.*

Related to Fig.6
