# Supplementary material for: Cell Marker Accordion: interpretable single-cell and spatial omics annotation in health and disease
Source: Nat Commun. 2025 Jul 7;16:5399. doi: 10.1038/s41467-025-60900-4 (PMC12234662; doi:10.1038/s41467-025-60900-4)
Supplement: Supplementary file 2 — Description of Additional Supplementary Files [file 41467_2025_60900_MOESM2_ESM.pdf]

Description of Additional Supplementary Files:

Supplementary Data 1: List of source gene marker databases, resources and integration procedures used to build the Cell Marker Accordion.

Supplementary Data 2: Comparison of available marker-based annotation tools in terms of features and capabilities.

Supplementary Data 3: Cell type specific annotation performances of single-cell and multi-omic datasets used for the benchmarking analysis.

Supplementary Data 4: List of all published datasets used in this study, including details on data processing, data availability and cell type mapping to Cell Ontology terms.

Supplementary Data 5: Tools and parameters used for the benchmarking analysis.

Supplementary Data 6: MDS patient characteristics and sequencing metrics.

Supplementary Data 7: Innate immune response gene signatures (from Gene Ontology).

Supplementary Data 8: The Cell Marker Accordion database.
