# Supplementary material for: Cell Marker Accordion: interpretable single-cell and spatial omics annotation in health and disease
Source: Nat Commun. 2025 Jul 7;16:5399. doi: 10.1038/s41467-025-60900-4 (PMC12234662; doi:10.1038/s41467-025-60900-4)
Supplement: Supplementary file 11 — Reporting Summary [file 41467_2025_60900_MOESM11_ESM.pdf]

nature portfolio

Corresponding author(s): Emma Busarello, Stephanie Halene, Toma Tebaldi

Last updated by author(s): May 14, 2025

Reporting Summary

Nature Portfolio wishes to improve the reproducibility of the work that we publish. This form provides structure for consistency and transparency in reporting. For further information on Nature Portfolio policies, see our Editorial Policies and the Editorial Policy Checklist.

Statistics

For all statistical analyses, confirm that the following items are present in the figure legend, table legend, main text, or Methods section.

n/a	Confirmed	
☐	☒	The exact sample size (*n*) for each experimental group/condition, given as a discrete number and unit of measurement
☐	☒	A statement on whether measurements were taken from distinct samples or whether the same sample was measured repeatedly
☐	☒	The statistical test(s) used AND whether they are one- or two-sided<br>*Only common tests should be described solely by name; describe more complex techniques in the Methods section.*
☒	☐	A description of all covariates tested
☐	☒	A description of any assumptions or corrections, such as tests of normality and adjustment for multiple comparisons
☐	☒	A full description of the statistical parameters including central tendency (e.g. means) or other basic estimates (e.g. regression coefficient) AND variation (e.g. standard deviation) or associated estimates of uncertainty (e.g. confidence intervals)
☐	☒	For null hypothesis testing, the test statistic (e.g. *F*, *t*, *r*) with confidence intervals, effect sizes, degrees of freedom and *P* value noted<br>*Give P values as exact values whenever suitable.*
☒	☐	For Bayesian analysis, information on the choice of priors and Markov chain Monte Carlo settings
☒	☐	For hierarchical and complex designs, identification of the appropriate level for tests and full reporting of outcomes
☒	☐	Estimates of effect sizes (e.g. Cohen's *d*, Pearson's *r*), indicating how they were calculated

Our web collection on statistics for biologists contains articles on many of the points above.

Software and code

Policy information about availability of computer code

Data collection	The Cell Marker Accordion gene marker database is available as an Excel file (Supplementary Data 8). Count matrices of patient samples generated by CellRanger v7.1.0, along with U2AF1 S34F mutation information from VarTrix v1.1.19, are available in Zenodo (10.5281/zenodo.15241428). Due to privacy regulations, raw sequencing files from patients are available upon request. All publicly available scRNA-seq and spatial datasets used in this study are listed in Supplementary Data 4, and the corresponding Seurat objects are deposited in Zenodo (10.5281/zenodo.15241428).
Data analysis	The Cell Marker Accordion R package, with code, tutorial and documentation, is available at https://github.com/TebaldiLab/cellmarkeraccordion under MIT license and deposited in Zenodo (10.5281/zenodo.15403792). The code of the Cell Marker Accordion Shiny app is available at https://github.com/TebaldiLab/shiny_cellmarkeraccordion under MIT license and deposited in Zenodo (10.5281/zenodo.15412280).

For manuscripts utilizing custom algorithms or software that are central to the research but not yet described in published literature, software must be made available to editors and reviewers. We strongly encourage code deposition in a community repository (e.g. GitHub). See the Nature Portfolio guidelines for submitting code & software for further information.

Data

Policy information about availability of data

All manuscripts must include a data availability statement. This statement should provide the following information, where applicable:

- Accession codes, unique identifiers, or web links for publicly available datasets
- A description of any restrictions on data availability
- For clinical datasets or third party data, please ensure that the statement adheres to our policy

The Cell Marker Accordion gene marker database is available as an Excel file (Supplementary Data 8). Count matrices of patient samples generated by CellRanger v7.1.0, along with U2AF1 S34F mutation information from VarTrix v1.1.19, are available in Zenodo (10.5281/zenodo.15241428). Due to privacy regulations, raw sequencing files from patients are available upon request. All publicly available scRNA-seq and spatial datasets used in this study are listed in Supplementary Data 4, and the corresponding Seurat objects are deposited in Zenodo (10.5281/zenodo.15241428).The Cell Marker Accordion code and documentation are described in the manuscript and available at this link: https://github.com/TebaldiLab/cellmarkeraccordion.

Research involving human participants, their data, or biological material

Policy information about studies with human participants or human data. See also policy information about sex, gender (identity/presentation), and sexual orientation and race, ethnicity and racism.

Reporting on sex and gender	Female n=2; male n=6.
Reporting on race, ethnicity, or other socially relevant groupings	No socially relevant groupings.
Population characteristics	Age = 49-89 y.o., median 72.
Recruitment	Human primary cells were obtained with patients' written consent.
Ethics oversight	Approval by the Yale University Human Investigation Committee.

Note that full information on the approval of the study protocol must also be provided in the manuscript.

Field-specific reporting

Please select the one below that is the best fit for your research. If you are not sure, read the appropriate sections before making your selection.

☒ Life sciences ☐ Behavioural & social sciences ☐ Ecological, evolutionary & environmental sciences

For a reference copy of the document with all sections, see nature.com/documents/nr-reporting-summary-flat.pdf

Life sciences study design

All studies must disclose on these points even when the disclosure is negative.

Sample size	We included in our cohort 8 patients, 5 patients without splicing factor mutations, 3 patients with U2AF1 S34F mutation.
Data exclusions	No data exclusions.
Replication	To assure reproducibility, at least 3 patient samples per condition were analyzed.
Randomization	Presence or absence of U2AF1 mutation.
Blinding	Blinding is not relevant in this study based on single cell transcriptome profiling of U2AF1-mut vs wildtype patients.

Reporting for specific materials, systems and methods

We require information from authors about some types of materials, experimental systems and methods used in many studies. Here, indicate whether each material, system or method listed is relevant to your study. If you are not sure if a list item applies to your research, read the appropriate section before selecting a response.

Materials & experimental systems

n/a	Involved in the study
☐	☒ Antibodies
☒	☐ Eukaryotic cell lines
☒	☐ Palaeontology and archaeology
☒	☐ Animals and other organisms
☒	☐ Clinical data
☒	☐ Dual use research of concern
☒	☐ Plants

Methods

n/a	Involved in the study
☒	☐ ChIP-seq
☐	☒ Flow cytometry
☒	☐ MRI-based neuroimaging

Antibodies

Antibodies used: Pacific Blue anti-human CD34 antibody (BioLegend, Cat 343512) for CD34+ blast isolation (1:100 in 500 ul)

Validation: Biancon G, et al. Mol Cell, 2022.

Plants

Seed stocks: *Report on the source of all seed stocks or other plant material used. If applicable, state the seed stock centre and catalogue number. If plant specimens were collected from the field, describe the collection location, date and sampling procedures.*

Novel plant genotypes: *Describe the methods by which all novel plant genotypes were produced. This includes those generated by transgenic approaches, gene editing, chemical/radiation-based mutagenesis and hybridization. For transgenic lines, describe the transformation method, the number of independent lines analyzed and the generation upon which experiments were performed. For gene-edited lines, describe the editor used, the endogenous sequence targeted for editing, the targeting guide RNA sequence (if applicable) and how the editor was applied.*

Authentication: *Describe any authentication procedures for each seed stock used or novel genotype generated. Describe any experiments used to assess the effect of a mutation and, where applicable, how potential secondary effects (e.g. second site T-DNA insertions, mosiacism, off-target gene editing) were examined.*

Flow Cytometry

Plots

Confirm that:

☐ The axis labels state the marker and fluorochrome used (e.g. CD4-FITC).

☐ The axis scales are clearly visible. Include numbers along axes only for bottom left plot of group (a 'group' is an analysis of identical markers).

☐ All plots are contour plots with outliers or pseudocolor plots.

☐ A numerical value for number of cells or percentage (with statistics) is provided.

Methodology

Sample preparation: *Describe the sample preparation, detailing the biological source of the cells and any tissue processing steps used.*

Instrument: *Identify the instrument used for data collection, specifying make and model number.*

Software: *Describe the software used to collect and analyze the flow cytometry data. For custom code that has been deposited into a community repository, provide accession details.*

Cell population abundance: *Describe the abundance of the relevant cell populations within post-sort fractions, providing details on the purity of the samples and how it was determined.*

Gating strategy: *Describe the gating strategy used for all relevant experiments, specifying the preliminary FSC/SSC gates of the starting cell population, indicating where boundaries between "positive" and "negative" staining cell populations are defined.*

☐ Tick this box to confirm that a figure exemplifying the gating strategy is provided in the Supplementary Information.
